# C‐X‐C chemokine receptor type 4 promotes tubular cell senescence and renal fibrosis through β‐catenin‐inhibited fatty acid oxidation

**DOI:** 10.1111/jcmm.18075

**Published:** 2024-01-11

**Authors:** Qinyu Wu, Qiurong Chen, Dan Xu, Xiaoxu Wang, Huiyun Ye, Xiaolong Li, Yabing Xiong, Jiemei Li, Shan Zhou, Jinhua Miao, Weiwei Shen, Youhua Liu, Hongxin Niu, Ying Tang, Lili Zhou

**Affiliations:** ^1^ State Key Laboratory of Organ Failure Research, National Clinical Research Center for Kidney Disease, Guangdong Provincial Institute of Nephrology, Guangdong Provincial Key Laboratory of Renal Failure Research, Division of Nephrology, Nanfang Hospital Southern Medical University Guangzhou China; ^2^ Department of Nephrology The Third Affiliated Hospital of Southern Medical University Guangzhou China; ^3^ Special Medical Service Center, Zhujiang Hospital Southern Medical University Guangzhou China

**Keywords:** cell senescence, CXCR4, fatty acid oxidation, renal fibrosis, β‐Catenin

## Abstract

The prevalence of chronic kidney disease (CKD) is highly increasing. Renal fibrosis is a common pathological feature in various CKD. Previous studies showed tubular cell senescence is highly involved in the pathogenesis of renal fibrosis. However, the inducers of tubular senescence and the underlying mechanisms have not been fully investigated. C‐X‐C motif chemokine receptor 4 (CXCR4), a G‐protein‐coupled seven‐span transmembrane receptor, increases renal fibrosis and plays an important role in tubular cell injury. Whereas, whether CXCR4 could induce tubular cell senescence and the detailed mechanisms have not studied yet. In this study, we adopted adriamycin nephropathy and 5/6 nephrectomy models, and cultured tubular cell line. Overexpression or knockdown of CXCR4 was obtained by injection of related plasmids. We identified CXCR4 increased in injury tubular cells. CXCR4 was expressed predominantly in renal tubular epithelial cells and co‐localized with adipose differentiation‐related protein (ADRP) as well as the senescence‐related protein P16^INK4A^. Furthermore, we found overexpression of CXCR4 greatly induced the activation of β‐catenin, while knockdown of CXCR4 inhibited it. We also found that CXCR4 inhibited fatty acid oxidation and triggered lipid deposition in tubular cells. To inhibit β‐catenin by ICG‐001, an inhibitor of β‐catenin, could significantly block CXCR4‐suppressed fatty acid oxidation. Taken together, our results indicate that CXCR4 is a key mediator in tubular cell senescence and renal fibrosis. CXCR4 promotes tubular cell senescence and renal fibrosis by inducing β‐catenin and inhibiting fatty acid metabolism. Our findings provide a new theory for tubular cell injury in renal fibrosis.

## 1 INTRODUCTION

Chronic kidney disease (CKD) is becoming a global health problem, which affects approximately 10%–15% of population worldwide.^1^, ^2^, ^3^ Renal fibrosis is the histological manifestation of CKD, commonly progressing into an irreversible process of end‐stage renal disease (ESRD).^4^, ^5^ However, the early discerning of CKD is a huge challenge with no typical clinical manifestations. Also, there is a blankness of the therapeutic strategies for CKD except for dialysis and kidney transplants.^6^ Hence, to understand the underlying mechanisms and identify therapeutic targets for renal fibrosis would be highly significant for discovering the treatment approaches for CKD.

Recent reports showed senescent cells greatly accumulate in kidneys in various CKDs.^7^, ^8^ Cell senescence in tubular cells is an early event in renal fibrosis and is highly associated with the progression of disease.^9^, ^10^ Cell senescence is driven by the activation of p53, p21 as well as P16^INK4A^, with a phenotype of permanent cell cycle arrest. Senescent cells are at active metabolism and result in the release of a group of proinflammatory and profibrotic factors, known as senescence‐associated secretory phenotype (SASP), including transforming growth factor‐β1 (TGF‐β1), interleukin‐1β (L‐1β) and interleukin 6 (IL‐6).^11^, ^12^, ^13^ However, the underlying mechanisms of cell senescence in renal tubular cells remain to be elucidated in detail.

The C‐X‐C motif chemokine receptor 4 (CXCR4), a CXCR family member specific to response to stromal cell‐derived factor 1α (SDF‐1α), plays a pivotal role in various physiological and pathological processes such as angiogenesis, stem cell trafficking and tumour metastasis.^14^, ^15^, ^16^ Reports have shown that CXCR4 is significantly upregulated after kidney injury, and the sustaining activation of it aggravates the fibrotic responses.^17^, ^18^, ^19^, ^20^ Our previous studies also found that CXCR4 promotes tubular cell injury and renal fibrosis via activating β‐catenin signalling.^21^, ^22^, ^23^

Wnt/β‐catenin signalling is an evolutionarily conserved pathway. However, Wnt/β‐catenin is reactivated in renal fibrosis and has been identified as a vital player in cellular senescence in renal tubular epithelial cells.^5^, ^24^, ^25^, ^26^ In our previous study, we found Wnt/β‐catenin could promote the senescence of tubular epithelial cells and age‐related renal fibrosis through inducing mitochondrial disturbances.^25^ However, the relationship between CXCR4 and cell senescence in renal fibrosis has not been demonstrated. Furthermore, whether CXCR4 affects mitochondrial dysfunction in tubular cells and the association of β‐catenin is still in mystery.

Renal tubular cells frequently execute the function of secretion and reabsorption. They are at a high baseline level of energy consumption. Fatty acid oxidation (FAO) is a primary source to meet their energy demands.^27^, ^28^ The regulation of lipid metabolism and FAO heavily depends on the activation of peroxisome proliferator–activated receptor α (PPARα).^29^ Loss of function of PPARα and defective FAO in tubular cells are not only involved in ATP depletion and lipid accumulation but also participate in cell death, dedifferentiation and fibrogenesis.^27^, ^30^, ^31^, ^32^ These observations prompt us to investigate the potential role of CXCR4 in FAO deficiency and renal tubular epithelial cell senescence.

In this study, we found CXCR4‐induced renal tubular epithelial cell senescence. CXCR4 also triggered FAO deficiency, cellular senescence and renal fibrosis and was linked to the activation of β‐catenin signalling. Targeted inhibition on the CXCR4/β‐catenin pathway could maintain fatty acid metabolism and retard renal fibrosis. Our results demonstrated that CXCR4 plays a crucial role in mediating tubular cell senescence and renal fibrosis by inducing β‐catenin‐triggered FAO deficiency. Hence, our findings reveal a new mechanism of renal fibrosis and suggest that targeted inhibition of CXCR4 provides a new therapeutic strategy for treating CKD.

## 2 MATERIALS AND METHODS

### 2.1 Animal models

Male BALB/c or CD‐1 mice (20–25 g), aged 7 weeks, were obtained from Southern Medical University Animal Center and were constructed for adriamycin nephropathy (ADR) as well as 5/6 nephrectomy (5/6NX) mice model, respectively. For ADR model, BALB/c mice were administered a single intravenous injection of adriamycin (doxorubicin hydrochloride, Sigma, St. Louis, MO) at 10.5 mg/kg body weight. Groups of mice (*n* = 5) were euthanized at 3 weeks after ADR injection and kidney tissues were collected for various analyses. For the 5/6NX model, male CD‐1 mice were removed off the upper and lower poles of the left kidney, two thirds of the left kidney and the right kidney 1 week later (week 0). The mice were killed at the end of week 5, and the remaining 1/6 kidney was used for the experimental test. The detailed experimental designs are shown in Figures 2A, 3A and 5A. Mouse CXCR4siRNA sequence (5′‐CGAUCAGUGUGAGUAUAUATT‐3′) was ligated into an shRNA expression plasmid (pLVX‐shRNA). Groups of mice were injected with the mouse CXCR4 expression plasmid (pFlag‐CXCR4) or shRNA expression plasmid (pLVX‐shCXCR4) by rapid injection of a large volume of DNA solution through the tail vein, as described previously.^21^ All matters related to animal experiments were authorized by the Animal Ethics Committee at the Nanfang Hospital, Southern Medical University.

### 2.2 Cell culture and treatment

Human proximal tubular epithelial cells (HK‐2) were obtained from the American Type Culture Collection (Manassas, VA) and maintained as routine protocol. HK‐2 cells were treated with SDF‐1α (SRP4388; Sigma‐Aldrich, St. Louis, MO). Some cells were transfected with empty vector (pcDNA3), CXCR4 expression plasmid (pFlag‐CXCR4) via JetPRIME® (Polyplus‐transfection S.A, Illkirch, France) followed by the stimulation of ICG‐001 (847591‐62‐2, Chemleader, Shanghai, China) at 10 μM. Then the whole‐cell lysates were prepared and subjected to western blot analyses or immunostaining. The detailed experimental designs and dosages were shown in figure legends, respectively.

### 2.3 Western blot analysis

Protein expression was detected by western blot analysis.^33^ The primary antibodies used were as follows: anti‐fibronectin (F3648; Sigma‐Aldrich), anti‐α‐SMA (A2547; Sigma‐Aldrich), anti‐CXCR4 (sc‐53,534; Santa), anti‐Snail1 (ab180714; Abcam), anti‐MMP‐7 (104,658; GTX), anti‐active β‐catenin (19,807 s; Cell Signalling Technology), anti‐P16^INK4A^ (ab241543; abcam), anti‐p19^ARF^ (ab202225; abcam), anti‐γH2AX (ab2893; abcam), anti‐CPT1A (ab128568; abcam), anti‐ACOX1 (A8091; ABclonal), anti‐PPARα (A18252; ABclonal), anti‐α‐tubulin (RM2007; Ray Antibody Biotech), anti‐GAPDH (RM2002; Ray Antibody Biotech) and anti‐β‐actin (RM001; Ray Antibody Biotech).

### 2.4 Histology and immunohistochemical staining

For immunohistochemical staining, 3‐μM‐thick paraffin sections of kidney samples were prepared. Periodic acid‐Schiff (PAS) and Sirius red staining were performed by a standard protocol or according to the manufacturer's instructions. Immunohistochemical staining was performed using routine protocol as described previously.^24^ Images were taken with an Olympus DP80 microscope with an EMCCD camera. Primary antibodies used were as follows: anti‐CXCR4 (PA1237; Boster), anti‐fibronectin (F3648; Sigma‐Aldrich), anti‐α‐SMA (A2547; Sigma‐Aldrich), anti‐γH2AX (A11463; ABclonal), anti‐P16^INK4A^ (sc‐1661; Santa) and anti‐β‐catenin (ab15180; abcam).

### 2.5 Immunofluorescence staining

HK‐2 cells cultured on coverslips were fixed with 4% paraformaldehyde for 15 min at room temperature, followed by permeabilizing with 0.2% of Triton X‐100 (T8787; Sigma‐Aldrich) for 10 min and blocking with 10% of donkey serum for 1 h. Paraffin‐embedded mouse kidney sections (3 μm thickness) were prepared by a routine procedure. Then the slides were immunostained with special primary antibodies overnight at 4°C. The primary antibodies included: anti‐fibronectin (F3648; Sigma‐Aldrich), anti‐Flag (M185‐3S, MBL), anti‐ADRP (ab52356; Abcam), β‐catenin (ab15180; abcam), anti‐CXCR4 (sc‐53,534; Santa), anti‐CPT1A (ab128568; abcam), anti‐Lotus Tetragonolobus Lectin (LTL) (FL‐1321; VECTOR Laboratories), anti‐Peanut Agglutinin (PNA) (FL‐1071; VECTOR Laboratories) and anti‐Aquaporin 3 (AQP3) (ab125219; abcam). After washing, slides were incubated with Cy2‐ or Cy3‐conjugated donkey anti‐mouse or anti‐rabbit IgG (Jackson Immuno‐Research Laboratories, West Grove, PA). Nuclei were stained with DAPI (C1006, Beyotime) according to the manufacturer's instructions. Images were taken by confocal microscopy (Leica TCS SP2 AOBS, Leica Microsystems, Buffalo Grove, IL).

### 2.6 SA‐β‐Gal staining

Frozen sections (3 μm) and coverslips of HK‐2 cells were assessed by the staining of senescence β‐galactosidase activity (#9860; Cell Signalling Technology). Briefly, frozen sections or coverslips were fixed for 15 min at room temperature and washed with PBS, then incubated in the SA‐β‐gal staining solution overnight at 37°C. Images were taken by an Olympus DP80 microscope with EMCCD camera.

### 2.7 Serum creatinine (Scr), blood urea nitrogen (BUN) and urinary albumin assay

Serum creatinine and blood urea nitrogen levels were determined by an automatic chemistry analyser (AU480 Chemistry Analyser, Beckman Coulter, Atlanta, Georgia). Urinary creatinine level was determined by use of a QuantiChrom creatinine assay kit (DICT‐500, Bioassay Systems, Hayward, CA), according to manufacturers' protocol. Urinary albumin was standardized to urine creatinine and expressed as mg/mg Ucr.

### 2.8 Blood pressure measurement

Tail‐cuff measurements of systolic (SBP) and mean blood pressures (MBP) were performed by a validated method that relies on volume pressure recording (VPR) technology (CODA8; Kent Scientific Corporation, Torrington, CT) and a radio‐telemetry system.^34^ In brief, before measurement, the mice were fixed comfortably and kept at rest in a tube‐shaped holder. The room temperature was set at 26°C. On the Kent warming panel, blood pressure measuring was repeated for nine times upon temperature at the base of tail reached 32°C. The normal measuring process was screened out by the blood measuring software system.

### 2.9 Transmission electron microscopy

Kidney cortex was collected and fixed in 1.25% glutaraldehyde/0.1 M phosphate buffer before detection. Ultrathin sections (60 nm) were prepared by a routine procedure and examined to assess kidney tubular mitochondrial ultrastructure as well as the accumulation of lipid droplets under an electron microscope (JEOL JEM‐1010).

### 2.10 Nile red staining

Frozen sections (6 μm) were fixed with 4% paraformaldehyde for 15 min at room temperature. Then the slides were incubated with a Nile red (Sigma 72,458) and DAPI (Sigma‐Aldrich) dual staining solution in the dark room for 10 min. Images were taken by confocal microscopy (Leica TCS SP2 AOBS, Leica Microsystems, Buffalo Grove, IL).

### 2.11 Statistical analyses

All data examined were expressed as mean ± SEM. Statistical analysis of the data was carried out using SPSS 19.0 (SPSS Inc, Chicago, IL). Comparisons were made by Student *t*‐test for comparison of two groups or via one‐way anova followed by the Least Significant Difference or Dunnett's T3 procedure for comparison of more than two groups. A value of *p* < 0.05 was considered statistically significant.

## 3 RESULTS

### 3.1 CXCR4 is upregulated in renal tubular cells and associated with FAO deficiency, cell senescence and β‐catenin activation

**FIGURE 1:**
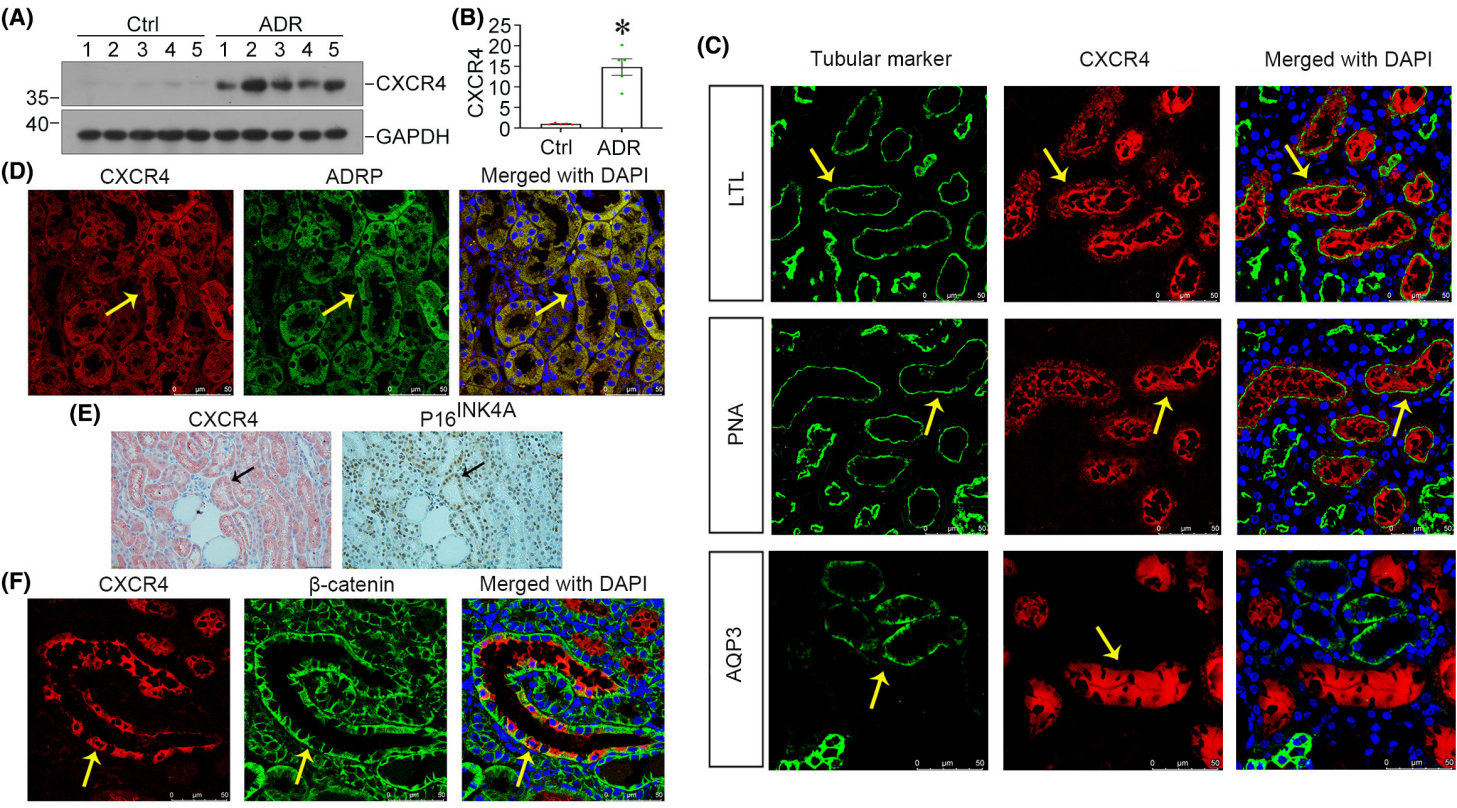
CXCR4 is upregulated in renal fibrosis and associated with FAO deficiency, RTECs senescence and β‐catenin activation. (A, B) Representative western blot (A) and quantitative data show the expression of CXCR4 (B) in two groups. *p < 0.05 versus normal controls (*n* = 5). (C) Co‐localization of CXCR4 and various segment‐specific tubular markers in kidneys from ADR mice model. Paraffin sections from ADR mice model as indicated were immunostained for CXCR4 (red) and various segment‐specific tubular markers (green). The following segment‐specific tubular markers were used: proximal tubule, lotus tetragonolobus lectin (LTL); distal tubule, peanut agglutinin (PNA); and collecting duct, aquaporin‐3 (AQP3). Arrows indicate positive tubules with co‐localization of CXCR4 and specific tubular markers. Scale bar: 50 μm. (D) Co‐localization of CXCR4 and ADRP in the tubules of ADR mice model. Kidney Paraffin sections from ADR mice model as indicated were immunostained for CXCR4 (red) and ADRP (green). Scale bar: 50 μm. (E) Co‐localization of CXCR4 and P16^INK4A^ in renal tubules of ADR mice model. Sequential paraffin‐embedded kidney sections from ADR mice model as indicated were immunostained for CXCR4 (left) and P16^INK4A^ (right). Arrows indicate positive staining. Scale bar: 50 μm. (F) Co‐localization of CXCR4 and β‐catenin in renal tubules of ADR mice model. Paraffin sections from ADR mice model as indicated were immunostained for CXCR4 (red) and β‐catenin (green). Co‐localization of CXCR4 and β‐catenin is indicated by arrows. Scale bar: 50 μm.

We first analysed the expression of CXCR4 in adriamycin nephropathy (ADR) mice. As shown in Figure 1A,B, CXCR4 is significantly increased in ADR mice. We then examined the tubular regional expression of CXCR4 in ADR mice. As shown in Figure 1C, CXCR4 was co‐localized with lotus tetragonolobus lectin (LTL), a marker of proximal tubules, and peanut agglutinin (PNA), a marker of distal tubules, but not with aquaporin‐3 (AQP3), a marker of collecting duct epithelium.^25^ We next carried out double staining for CXCR4 with adipose differentiation‐related protein (ADRP), a major lipid droplet‐associated protein.^35^ As shown, CXCR4 was nearly completely co‐localized with ADRP in renal tubular epithelium (Figure 1D). We then examined the co‐localization of CXCR4 and P16^INK4A^, a cell senescence marker,^24^ in sequential paraffin‐embedded kidney sections. As shown in Figure 1E, CXCR4 was also co‐localized with P16^INK4A^ in tubules. Furthermore, double staining showed that CXCR4 was co‐localized with β‐catenin (Figure 1F). These results suggest that CXCR4 is majorly upregulated in renal tubular cells and highly involved in renal tubular cell senescence and lipid metabolism disorders, and the mechanisms are associated with β‐catenin activation.

### 3.2 Ectopic CXCR4 induces renal fibrosis through disturbing fatty acid metabolism and aggravating tubular senescence

**FIGURE 2:**
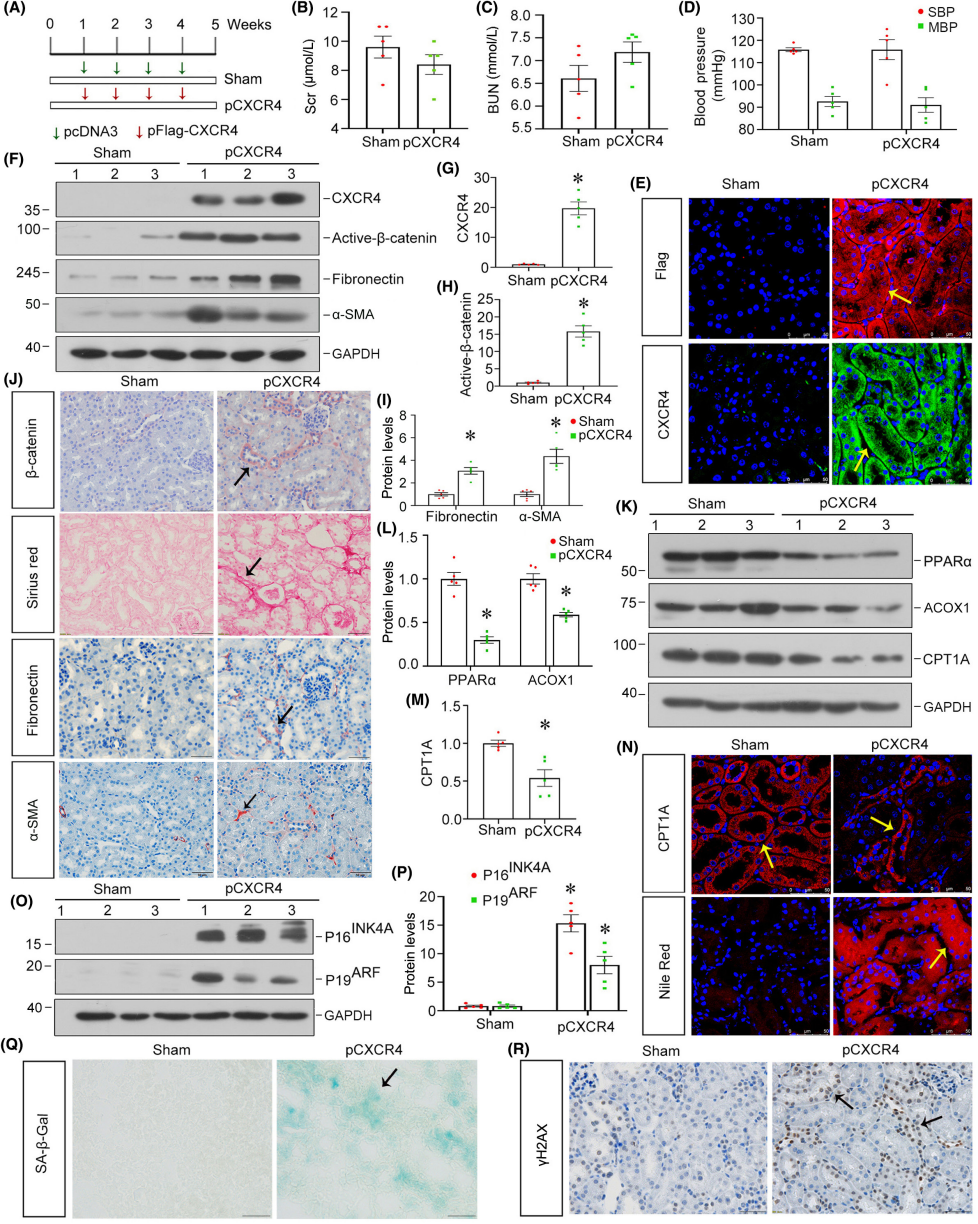
Ectopic expression of CXCR4 induces renal fibrosis through disturbing renal fatty acid metabolism and aggravating tubular senescence in normal mice. (A) Experimental design. Green arrows indicate the intravenous injections of empty vector pcDNA3, and red arrows indicate the intravenous injections of pFlag‐CXCR4 for 4 weeks in normal mice. (B, C) Scr and BUN were measured in indicated groups. No significance versus normal controls (*n* = 5). (D) Graphical presentation shows blood pressure in two groups. No significance versus normal controls (*n* = 5). (E) Representative micrographs show the expression of Flag and CXCR4 in two groups. Paraffin sections were stained with different antibodies. Arrows indicate positive staining. Scale bars: 50 μm. (F–I) Representative western blot (F) and quantitative data show the expression of CXCR4 (G), active‐β‐catenin (H) and fibronectin and α‐SMA (I) in two groups. *p < 0.05 versus normal controls (*n* = 5). (J) Representative micrographs show the expression of β‐catenin and fibronectin and α‐SMA as well as Sirius red staining in two groups. Arrows indicate positive staining. Scale bars: 50 μm. (K–M) Representative western blot (K) and quantitative data for PPARα, ACOX1 (L) and CPT1A (M) in two groups. *p < 0.05 versus normal controls (*n* = 5). (N) Representative micrographs show the expression of CPT1A and the formation of lipids by Nile Red staining in two groups. Paraffin sections were stained with CPT1A. Frozen sections were stained with Nile red. Arrows indicate positive staining. Scale bars: 50 μm. (O, P) Representative western blot (O) and quantitative data for P16^INK4A^ and p19^ARF^ (P) in two groups. *p < 0.05 versus normal controls (*n* = 5). (Q) Representative micrographs showing renal expression of SA‐β‐gal activity in two groups. Frozen kidney sections were stained for SA‐β‐gal activity. Arrows indicate positive staining. Scale bars: 50 μm. (R) Representative micrographs showing renal expression of γH2AX in two groups. Paraffin‐embedded kidney sections were immunostained with an antibody against γH2AX. Arrows indicate positive staining. Scale bars: 50 μm.

To examine the role of CXCR4 in renal fibrosis, we delivered a flag‐tagged expression plasmid (pFlag‐CXCR4) into normal mice via a hydrodynamic‐based approach.^21^ Experimental designs are shown in Figure 2A. As shown, ectopic CXCR4 did not affect serum creatinine (Scr), blood urea nitrogen (BUN) levels as well as blood pressure (Figure 2B–D). The immunostaining of Flag and CXCR4 confirmed that CXCR4 was successfully delivered into renal tubules (Figure 2E). Western blot showed that ectopic CXCR4 induced the expression of active β‐catenin as well as fibronectin and α‐SMA, two fibrosis‐related proteins (Figure 2F–I). Similar results were observed when β‐catenin, fibronectin and α‐SMA were analysed by immunostaining (Figure 2J). Sirius red staining also revealed that CXCR4‐induced renal interstitial fibrosis (Figure 2J). All these data suggested that overexpression of CXCR4 could mediate β‐catenin activation and induce fibrotic lesions.

We then assessed the role of CXCR4 on FAO deficiency and cell senescence. As shown in Figure 2K–M, western blot showed that the expression of peroxisome proliferator‐activated receptor (PPARα), carnitine palmitoyl transferase‐1A (CPT1A) and Acyl‐CoA oxidase‐1 (ACOX1), the three key FAO regulators,^36^ was inhibited after ectopic expression of CXCR4. Similar result was obtained when CPT1A was analysed by immunofluorescence staining (Figure 2N). Nile red staining also showed that CXCR4 induced the accumulation of lipids (Figure 2N). We further analysed the effects of CXCR4 on cell senescence. As shown in Figure 2O,P, ectopic CXCR4 upregulated the expression of P16^INK4A^ and P19^ARF^, two senescence markers.^37^ We also tested the cellular senescence by senescence‐associated β‐galactosidase (SA‐β‐gal) staining.^38^ As shown, tubular senescence, which appeared in the bright‐blue granular staining, was greatly induced by overexpression of CXCR4 (Figure 2Q). Similar result was observed when γH2AX, an important factor presenting senescent pathway,^24^ was assessed by western blot (Figure 2R). These results indicated that overexpression of CXCR4 elicited lipid accumulation, inhibited fatty acid oxidation and accelerated tubular cell senescence.

### 3.3 Blockade of CXCR4 alleviates renal fibrosis in ADR nephropathy

**FIGURE 3:**
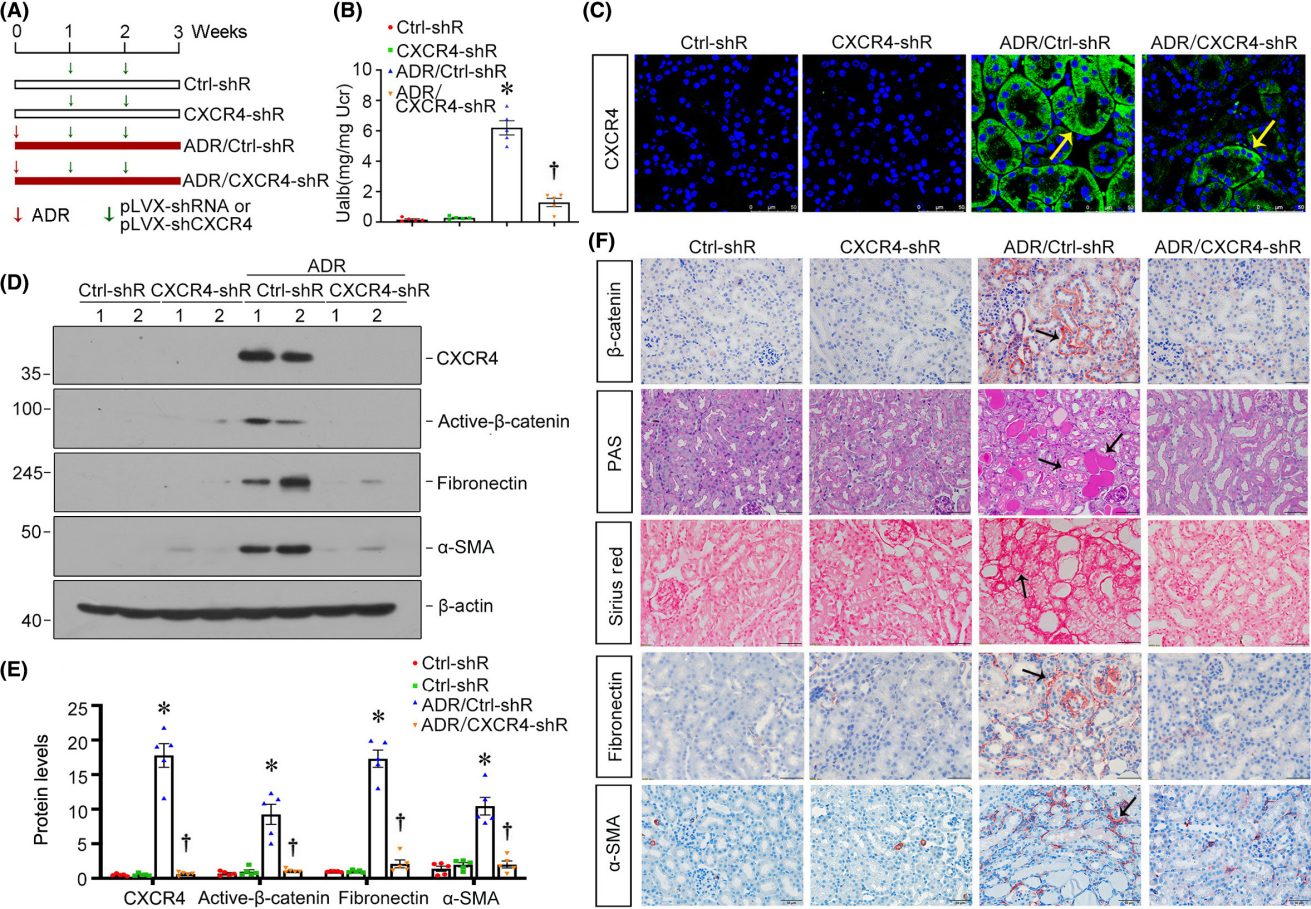
Blockade of CXCR4 alleviates renal fibrosis in ADR nephropathy. (A) Experimental design. Green arrows indicate the intravenous injections of either an empty vector (pLVX‐shRNA) or an shRNA vector encoding the interference sequence of CXCR4 (pLVX‐shCXCR4). The red bar indicates the timing of administration of Adriamycin. (B) Urinary albumin (Ualb) was measured in different groups. *p < 0.05 versus normal controls; †*p* < 0.05 versus ADR alone (*n* = 5). (C) Representative micrographs show the expression of CXCR4 in four groups. Paraffin sections were stained with an antibody against CXCR4. Arrows indicate positive staining. Scale bars: 50 μm. (D, E) Representative western blot (D) and quantitative data show the expression of CXCR4, active‐β‐catenin, fibronectin and α‐SMA (E) in four groups. *p < 0.05 versus normal controls; †*p* < 0.05 versus ADR alone (*n* = 5). (F) Representative micrographs show the PAS and Sirius red staining as well as the expression of β‐catenin, fibronectin and α‐SMA in four groups. Arrows indicate positive staining. Scale bars: 50 μm.

To block renal CXCR4 expression, we then injected an shRNA vector encoding the interference sequence of CXCR4 (pLVX‐shCXCR4) into adriamycin nephropathy (ADR) mice. The experimental design is shown in Figure 3A. We first analysed albuminuria levels, one of the markers defining kidney function.^39^ As shown, blockade of CXCR4 significantly decreased the level of urinary albumin (Ualb) in ADR mice, but not in normal mice (Figure 3B). The efficacy of CXCR4 blockade was confirmed by immunostaining of CXCR4, and the western blot also showed that the expression of CXCR4 was successfully interfered in the kidney (Figure 3C,D). We then examined the expression of active β‐catenin, fibronectin and α‐SMA. As shown in Figure 3D,E, the expressional levels of active β‐catenin, fibronectin and α‐SMA were induced in ADR mice, but significantly diminished by interference of CXCR4. Consistently, similar results were obtained when active β‐catenin, fibronectin and α‐SMA were assessed by immunostaining (Figure 3F). Periodic acid‐Schiff (PAS) and Sirius red staining also indicated that blockade of CXCR4 relieved renal tubular cell injury and interstitial fibrosis (Figure 3F). Consistent with our previous study,^21^ our results demonstrated that blockade of CXCR4 alleviated renal fibrosis through inhibiting β‐catenin activation.

### 3.4 Blockade of CXCR4 restores fatty acid metabolism and relieves tubular senescence in ADR nephropathy

**FIGURE 4:**
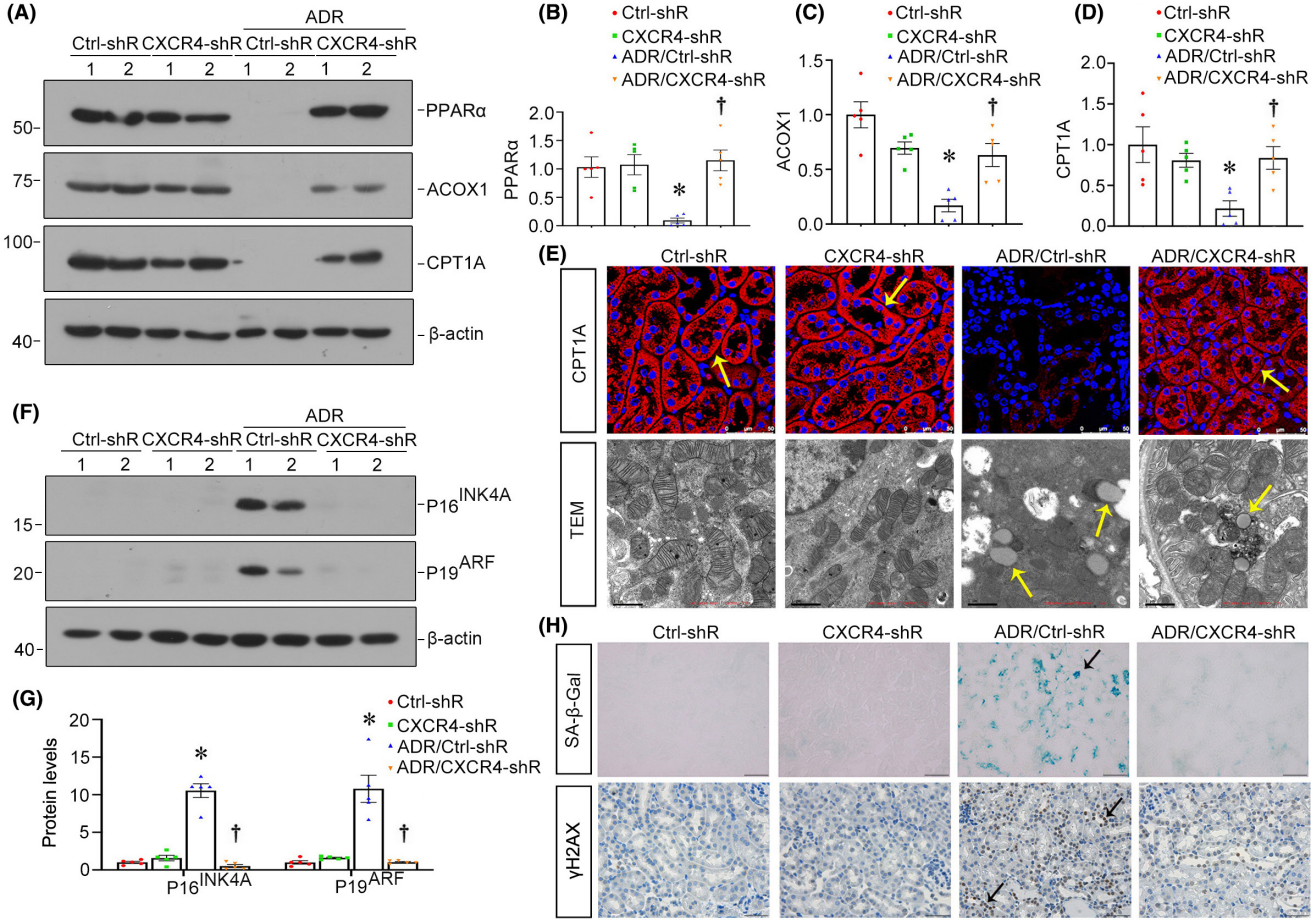
Blockade of CXCR4 restores fatty acid metabolism and relieves tubular senescence in ADR nephropathy. (A–D) Representative western blot (A) and quantitative data for PPARα (B), ACOX1 (C) and CPT1A (D) in different groups. *p < 0.05 versus normal controls (*n* = 5); †*p* < 0.05 versus ADR alone (*n* = 5). (E) Representative micrographs show the expression of CPT1A in different groups. Paraffin sections were stained with CPT1A. Arrows indicate positive staining. Scale bars: 50 μm. Representative transmission electron microscopy (TEM) micrographs show lipid droplets in ultrathin kidney sections. Scale bars: 1 μm. (F) Representative western blot (F) and quantitative data for P16^INK4A^ and p19^ARF^ (G) in different groups. *p < 0.05 versus normal controls; †*p* < 0.05 versus ADR alone (*n* = 5). (H) Representative micrographs showing renal SA‐β‐gal activity and the expression of γH2AX in different groups. Frozen kidney sections were stained for SA‐β‐gal activity. Paraffin‐embedded kidney sections were immunostained with an antibody against γH2AX. Arrows indicate positive staining. Scale bars: 50 μm.

We next examined the effect of CXCR4 blockade on FAO metabolism and cell senescence. As shown in Figure 4A–D, PPARα, ACOX1 and CPT1A were downregulated in ADR mice, while blockade of CXCR4 significantly restored their expression. A similar result was found when CPT1A was tested by immunostaining. Transmission electron microscopy (TEM) also showed the accumulation of lipid droplets in ADR mice, which was greatly reduced by interference of CXCR4 (Figure 4E). We then tested cellular senescence by examining the protein levels of P16^INK4A^ and P19^ARF^. As shown in Figure 4F,G, P16^INK4A^ and P19^ARF^ were induced in ADR mice but significantly decreased by blockade of CXCR4. Similar results were observed when SA‐β‐gal activity and γ‐H2AX were assessed by staining (Figure 4H). These results indicated that blockade of CXCR4 restores fatty acid metabolism and relieves tubular senescence.

### 3.5 Ectopic CXCR4 promotes renal fibrosis and is accompanied by activation of β‐catenin in 5/6NX mice

**FIGURE 5:**
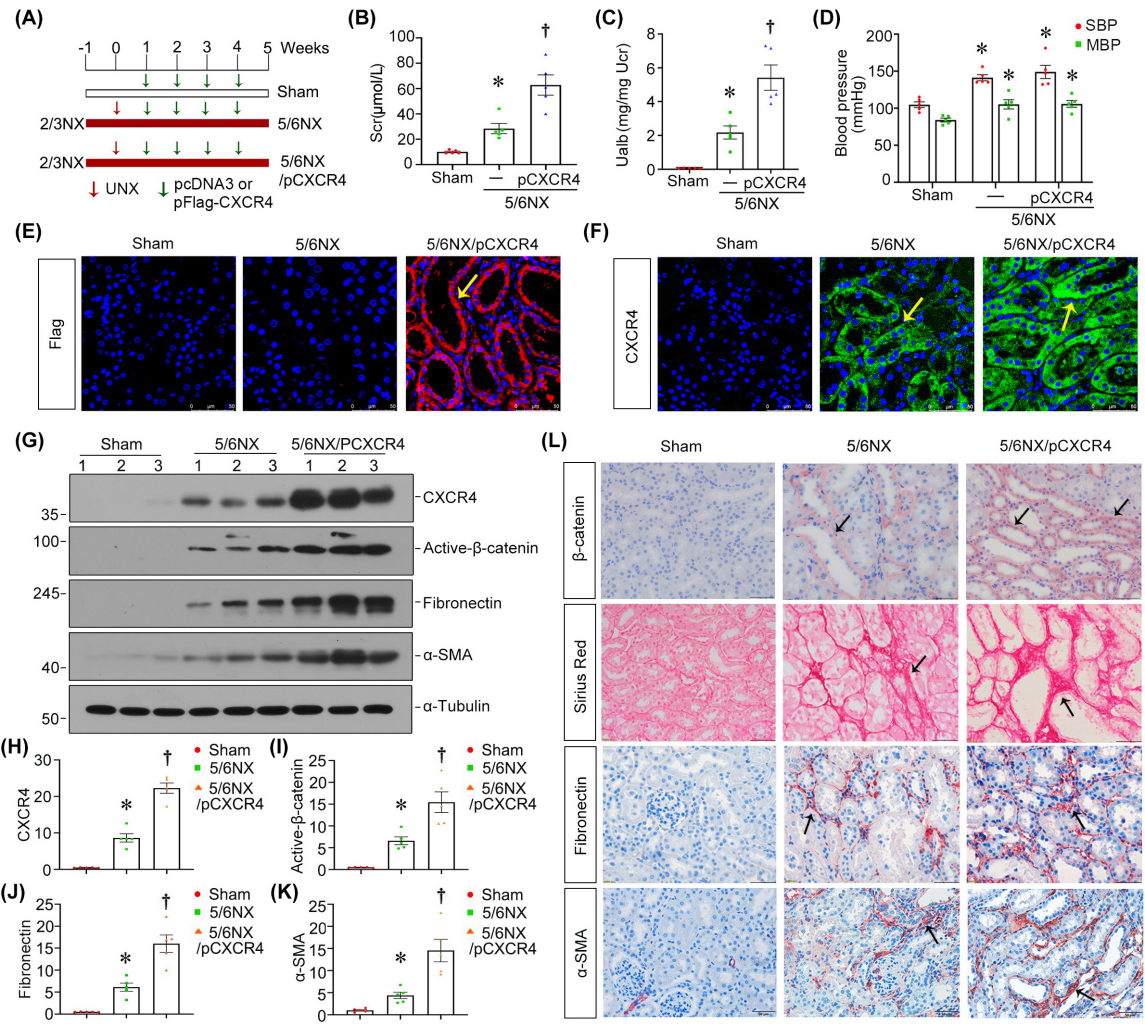
Ectopic CXCR4 promotes renal fibrosis and is accompanied by activation of β‐catenin in 5/6NX mice. (A) Experimental design. Green arrows indicate the intravenous injections of either empty vector pcDNA3 or pFlag‐CXCR4 at 1, 2, 3 and 4 weeks after 5/6 NX. The red arrows indicate unilateral nephrectomy. (B, C) Scr and Urinary albumin (Ualb) were measured in different groups. *p < 0.05 versus sham controls; †*p* < 0.05 versus 5/6 NX alone (*n* = 5). (D) Graphical presentation shows blood pressure in three groups. *p < 0.05 versus sham controls (*n* = 5). (E) Representative micrographs show the expression of Flag in different groups. Paraffin sections were stained with Flag. Arrows indicate positive staining. Scale bars: 50 μm. (F) Representative micrographs show the expression of CXCR4 in different groups. Paraffin sections were stained with CXCR4. Arrows indicate positive staining. Scale bars: 50 μm. (G–K) Representative western blot (G) and quantitative data for CXCR4 (H), active‐β‐catenin (I), fibronectin (J) and α‐SMA (K) in different groups. *p < 0.05 versus sham controls; †*p* < 0.05 versus 5/6 NX alone (*n* = 5). (L) Representative micrographs show the expression of β‐catenin, fibronectin, α‐SMA as well as Sirius red staining in three groups. Arrows indicate positive staining. Scale bars: 50 μm.

We further delivered pFlag‐CXCR4 plasmid into 5/6NX mice. Experimental design is shown in Figure 5A. As shown in Figure 5B,C, the levels of Scr and Ualb, markers of kidney function,^40^ were increased in 5/6NX mice, and further aggravated by ectopic CXCR4. However, CXCR4 could not further elevate the blood pressure in 5/6NX mice (Figure 5D). Immunostaining of Flag and CXCR4 confirmed that CXCR4 was successfully delivered into the kidney (Figure 5E,F). As shown in Figure 5G–L, ectopic CXCR4 further aggravated the activation of β‐catenin and induced renal fibrosis. These results further strongly suggested that CXCR4 plays a critical role in renal fibrosis by inducing β‐catenin activation in 5/6NX mice.

### 3.6 Ectopic CXCR4 inhibits fatty acid metabolism and accelerates cell senescence in renal tubular cells in 5/6NX mice

**FIGURE 6:**
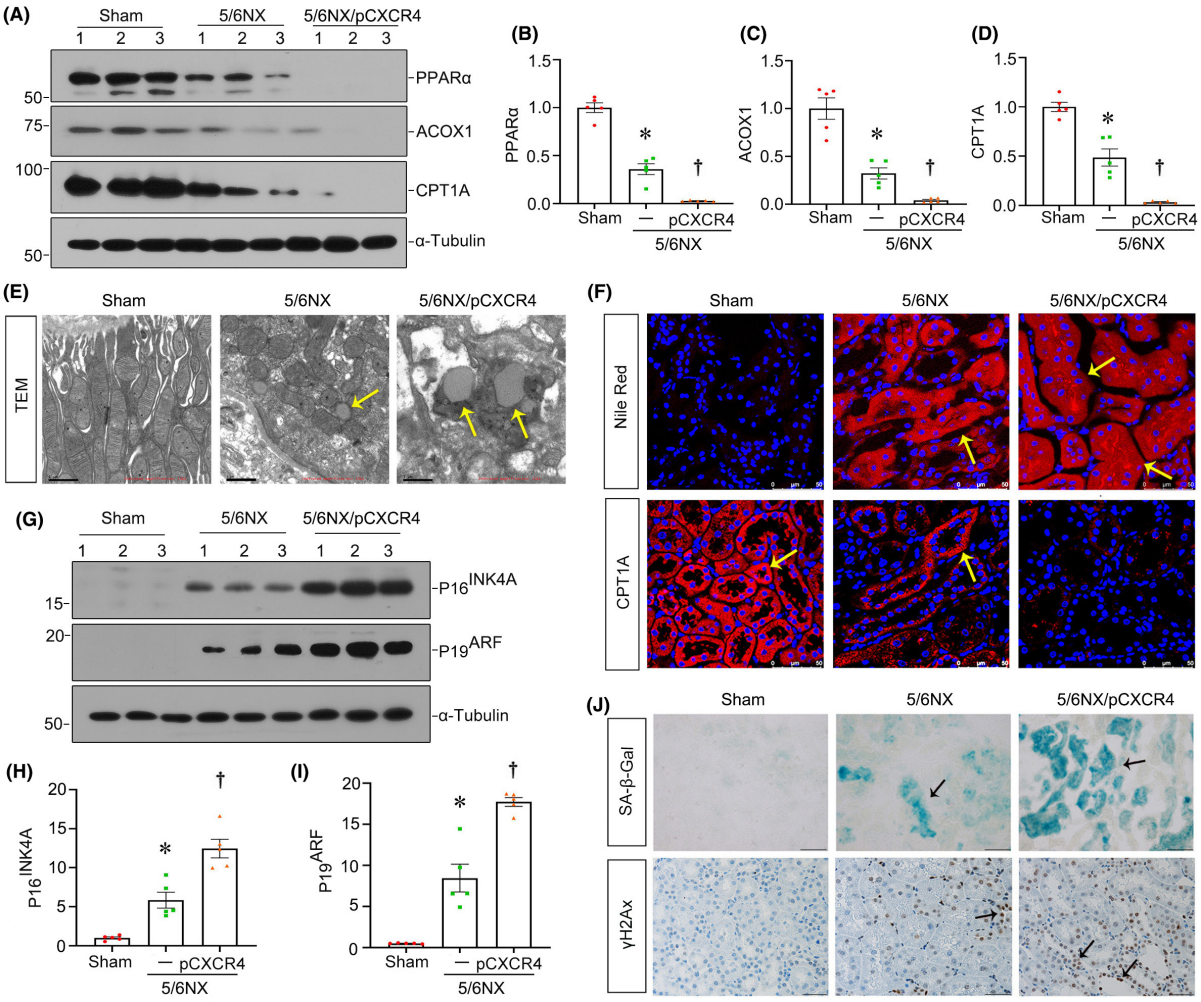
Ectopic CXCR4 inhibits fatty acid metabolism and accelerates senescence of RTECs in 5/6NX. (A–D) Representative western blot (A) and quantitative data for PPARα (B), ACOX1 (C) and CPT1A (D) in different groups. *p < 0.05 versus sham controls; †*p* < 0.05 versus 5/6 NX alone (*n* = 5). (E) Representative transmission electron microscopy (TEM) micrographs show lipid droplets in ultrathin kidney sections in different groups. Scale bars: 1 μm. (F) Representative micrographs show the Nile red staining and the expression of CPT1A in different groups. Frozen kidney sections were stained with Nile red, paraffin sections were stained with CPT1A. Scale bars: 50 μm. (G–I) Representative western blot (G) and quantitative data for P16^INK4A^ (H) and p19^ARF^ (I) in different groups. *p < 0.05 versus sham controls; †*p* < 0.05 versus 5/6 NX alone (*n* = 5). (J) Representative micrographs showing renal SA‐β‐gal activity and the expression of γH2AX in different groups. Frozen kidney sections were stained for SA‐β‐gal activity. Paraffin‐embedded kidney sections were immunostained with an antibody against γH2AX. Arrows indicate positive staining. Scale bars: 50 μm.

We then assessed FAO deficiency and tubular cell senescence in 5/6NX mice. As shown in Figure 6A–D, the expression of PPARα, ACOX1 and CPT1A were decreased in 5/6NX mice and further aggravated by ectopic CXCR4. A similar result was obtained when CPT1A was detected by immunofluorescence staining (Figure 6F). Furthermore, TEM and Nile Red staining also revealed that CXCR4 exacerbated lipid accumulation in 5/6NX mice (Figure 6E,F). Next, we examined cellular senescence by assay of P16^INK4A^, P19^ARF^ and staining of SA‐β‐gal and γ‐H2AX. As shown in Figure 6G–J, ectopic expression of CXCR4 aggravated senescence in renal tubular epithelial cells in 5/6NX mice. All these results suggested that CXCR4 inhibits fatty acid metabolism and accelerates tubular cell senescence.

### 3.7 Inhibition of β‐catenin retards CXCR4‐induced FAO disorder, cellular senescence and renal fibrosis

**FIGURE 7:**
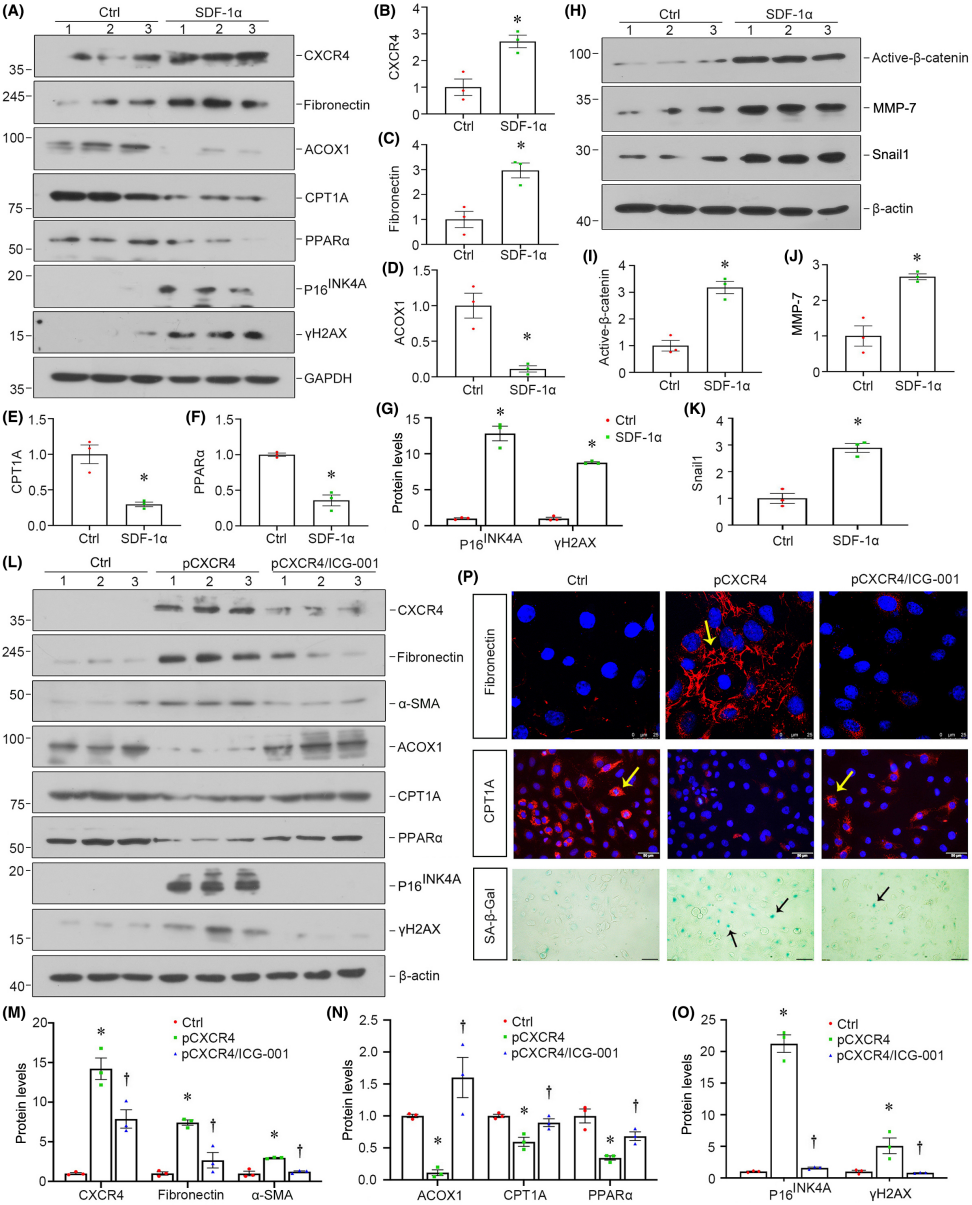
Inhibition of β‐catenin retards CXCR4‐induced disorder of FAO, cellular senescence and fibrosis. (A–G) Representative western blot (A) and quantitative data for CXCR4 (B), fibronectin (C), ACOX1 (D), CPT1A (E), PPARα (F), P16^INK4A^ and γH2AX (G) expression in two groups as indicated. HK‐2 cells were treated with SDF‐1α (100 ng/mL) for 48 h. *p < 0.05 versus control group (*n* = 3). (H–K) Representative western blot (h) and quantitative data for active‐β‐catenin (I), MMP‐7 (J) and Snail1 (K) expression in two groups as indicated. HK‐2 cells were treated with SDF‐1α (100 ng/mL) for 24 h. *p < 0.05 versus control group (*n* = 3). (L–O) Representative western blot (L) and quantitative data for CXCR4, fibronectin and α‐SMA (M), ACOX1, CPT1A and PPARα (N), P16^INK4A^ and γH2AX (O) expression in two groups as indicated. HK‐2 cells were pretreated with ICG‐001 (10 μM) and then transfected with pFlag‐CXCR4 or pcDNA3 plasmid for 48 h. *p < 0.05 versus pcDNA3 group (*n* = 3); †*p* < 0.05 versus pCXCR4 (pFlag‐CXCR4) alone (*n* = 3). (P) Representative micrographs showing the expression of fibronectin and CPT1A in HK‐2 cells. HK‐2 cells cultured on coverslips were immunostained with antibodies against fibronectin and CPT1A. Arrows indicate positive staining. Representative micrographs showing SA‐β‐gal activity in different groups. Arrows indicate positive staining. Scale bars: 50 μm. *p < 0.05 versus pcDNA3 group (*n* = 3); †*p* < 0.05 versus pCXCR4 (pFlag‐CXCR4) alone (*n* = 3).

We further examined the effect of CXCR4 in fatty acid metabolism and tubular cell senescence in vitro. The human proximal tubular epithelial cell line (HK‐2) was cultured and treated with SDF‐1α. As shown in Figure 7A–G, administration of SDF‐1α significantly triggered the expression of CXCR4, fibronectin, senescence‐related P16^INK4A^, γ‐H2AX and reduced the expression of ACOX1, CPT1A and PPARα. Meanwhile, SDF‐1α strongly induced the expression of active‐β‐catenin and its downstream target genes, such as MMP‐7 and Snail1, as shown by western blot (Figure 7H–K).

To further confirm the role of β‐catenin in CXCR4‐induced renal fibrosis, we pretreated HK‐2 cells with ICG‐001, a small molecule inhibiting β‐catenin‐mediated gene transcription,^41^ and then transfected cells with pFlag‐CXCR4 plasmid. Western blot analyses indicated that ICG‐001 pretreatment greatly restrained CXCR4‐induced expression of fibronectin, α‐SMA, P16^INK4A^ and γ‐H2AX as well as a decrease in PPARα, ACOX1, CPT1A and PPARα (Figure 7L–O). Similar results were observed when fibronectin, CPT1A and SA‐β‐gal activity were analysed by staining (Figure 7P). These results further clarify that CXCR4 plays a vital role in FAO deficiency, cellular senescence and fibrotic lesions. β‐catenin is an important downstream effector to mediate CXCR4 signalling.

## 4 DISCUSSION

CKD, with a high morbidity and mortality nowadays, is becoming a public health problem, which further leads to other complications such as cardiovascular diseases.^2^ Renal fibrosis, known as the most prominent mark of CKD, is characterized by tubular atrophy and excessive extracellular matrix (ECM) accumulation.^42^, ^43^ Of note, CKD exhibits many characteristics as age‐related lesions, including glomerular sclerosis, interstitial fibrosis, heightened levels of oxidative stress, and ongoing inflammation.^44^, ^45^ Emerging evidence suggest that senescent tubular cells could be highly detected in different types of CKD, and are positively correlated with the fibrotic injury and deterioration of kidney function.^24^, ^46^ Senescent tubular cells have strong impacts on surrounding cells by secreting various SASP molecules, including inflammatory cytokines, chemokines, growth factors and extracellular matrix remodelling factors, such as TGF‐β, plasminogen activator inhibitor‐1 (PAI‐1), monocyte chemoattractant protein‐1 (MCP‐1), interleukin 1α (IL‐1α) and interleukin‐6 (IL‐6), etc.^47^, ^48^, ^49^, ^50^ These suggest that senescent tubular cells play a crucial role in the development of renal fibrosis. Hence, to clarify the mechanisms of tubular cell senescence in renal fibrosis is especially important to open up a novel avenue to treat CKD.

The kidney is one of the most energy‐demanding organs in the body.^51^ Tubular epithelial cells are highly specialized cells that heavily rely on fatty acid oxidation (FAO) to accomplish their adenosine triphosphate (ATP) supply. Recently, many researchers have explored the significant role of impaired FAO pathways in the progression of renal fibrosis.^27^, ^30^, ^52^ Indeed, FAO metabolism deficiency has been shown to trigger epithelial–mesenchymal transition (EMT), inflammation, and interstitial fibrosis.^27^, ^32^ Clinical evidence has demonstrated that lipid droplets accumulate in renal tubular cells in patients with CKD,^53^ and high‐fat diet mice model.^54^ Some Reports indicate that there is a significant decrease in the expression of PPARα and the FAO pathway–associated proteins, accompanied by the accumulation of lipids in the renal tubular epithelial cells. Furthermore, PPARα^−/−^ mice exhibited an exaggerated lipid accumulation, a decreased activity of the FAO function and severe fibrotic lesions.^30^ These findings reveal that the damage of renal tubular cells is related to changes in lipid metabolism. However, the underlying mechanisms of FAO deficiency in renal fibrosis have not been elucidated in detail. Interestingly, in this study, we found that CXCR4 appears to play a central role in tubular cell senescence through activating β‐catenin‐mediated fatty acid metabolism deficiency.

CXCR4 was recently found to be primarily upregulated in tubular epithelial cells in renal fibrosis model and promoted kidney fibrosis by activating JAK/STAT/GSK3β/β‐catenin pathway, as reported by our previous work.^21^ In this study, we further confirmed that CXCR4 was predominantly expressed in proximal and distal tubules in renal fibrosis. Meanwhile, CXCR4 is highly co‐localized with ADRP, a key protein expressed in lipid droplets, as well as P16^INK4A^, a senescence marker. The results from staining of sequential sections support the notion that CXCR4 and β‐catenin are co‐localized in tubular cells, suggesting the intimate correlation between activation of CXCR4 and FAO deficiency, as well as cellular senescence induced by β‐catenin in kidney injury (Figure 1).

Subsequently, we performed the analysis in both experimental mouse models and cultured tubular cells. In normal and 5/6NX mice, ectopic CXCR4 activated β‐catenin signalling (Figures 2 and 5). This cascade of phosphorylation of signalling proteins is also consistent with our previous findings in other classic CKD models.^21^, ^22^ Meanwhile, CXCR4 triggered renal tubular cell senescence, renal fibrotic lesions and FAO deficiency (Figures 2, 5 and 6). Knockdown of CXCR4 in ADR mice model ameliorated renal injuries, and inhibited β‐catenin (Figure 3), preserved mitochondrial function and retarded cellular senescence (Figure 4). The inductive role of CXCR4 in tubular cell senescence was further confirmed in vitro. SDF‐1α, the ligand of CXCR4, significantly inhibited the enzymes and regulators of FAO exacerbated senescence‐related fibrotic lesions and evidently induced the activation of β‐catenin signalling. It is noteworthy that ICG‐001, a small molecule inhibitor of β‐catenin activation, effectively prevented CXCR4‐induced cell senescence and FAO deficiency in tubular cells (Figure 7). Collectively, these results revealed the central role of CXCR4/β‐catenin in renal tubular FAO impairment and cellular ageing.

Our study firstly indicates that CXCR4 promotes senescence‐associated renal fibrosis, which is mediated by β‐catenin activation. There are several studies indicated that the Wnt/β‐catenin pathway could induce senescence. It was reported from our laboratory that Wnt9a could induce senescent tubular cells to secrete TGF‐β1, a component of the SASP component, which in turn promotes EMT and subsequent fibrosis.^24^, ^25^ α‐Klotho, which is an endogenous antagonist of Wnt/β‐catenin signalling, has been found to significantly reverse Wnt‐induced mitochondrial injury, cellular senescence and fibrotic lesions.^5^, ^34^, ^55^ Furthermore, tubular aberrant expression of Brahma‐related gene 1 (BRG1), an enzymatic subunit of the SWItch/Sucrose non‐Fermentable complex, could trigger tubular senescence and renal fibrosis. This process is mediated by the activation of the Wnt/β‐catenin/autophagy axis.^56^ These observations suggest that Wnt/β‐catenin signalling activation could mediate senescence‐associated renal fibrosis, and CXCR4 could largely aggravate these effects.

Additionally, we found that β‐catenin participates in CXCR4‐induced FAO disorders and cell senescence. In in vitro study, inhibition of β‐catenin by ICG‐001 largely retarded CXCR4‐induced deficiency of FAO, cellular senescence and renal fibrosis. Likely, it has been confirmed that CXCR4 as well as the Wnt/β‐catenin pathway could regulate fatty acid metabolism in many CKDs, respectively.^57^, ^58^, ^59^ Recent studies have uncovered that tubular STAT6, a downstream gene of CXCR4, could promote lipid accumulation and renal fibrosis by suppressing FAO in the UUO model through a sis‐inducible element situated in the promoter region of the protein.^29^ Kidney injury molecule (KIM)‐1, known as targeted gene of β‐catenin, may be involved in the uptake of fatty acid by renal tubular cells, and lead to the progression of diabetic kidney diseases.^60^ All these observations support our findings that CXCR4 may regulate FAO deficiency and induce lipid accumulation via β‐catenin activation.

In summary, our findings first uncovered the key role of CXCR4 in regulating PPARα‐mediated fatty acid oxidation and renal tubular cell senescence‐related renal fibrosis through activating β‐catenin signalling. Given the rising prevalence of the elderly population and limited treatment options for CKD, to find a new target against cell senescence is significant to therapeutic strategies of CKD. This study provides a new insight into the role of CXCR4 in CKD. The targeted inhibition of CXCR4, whether by its antagonists or delivered gene therapy, would be a new and effective therapeutic strategy for preventing renal fibrosis.

## AUTHOR CONTRIBUTIONS

**Qinyu Wu:** Data curation (equal); formal analysis (equal); investigation (equal); methodology (equal); project administration (equal); validation (equal); visualization (equal); writing – original draft (equal). **Qiurong Chen:** Data curation (equal); formal analysis (equal); project administration (equal); writing – original draft (equal). **Dan Xu:** Data curation (equal); formal analysis (equal); methodology (equal); project administration (equal); visualization (equal). **Xiaoxu Wang:** Project administration (supporting). **Huiyun Ye:** Project administration (supporting). **Xiaolong Li:** Project administration (supporting). **Yabing Xiong:** Project administration (supporting). **Jiemei Li:** Project administration (supporting). **Shan Zhou:** Project administration (supporting). **Jinhua Miao:** Project administration (supporting). **Weiwei Shen:** Project administration (supporting). **Youhua Liu:** Conceptualization (supporting); supervision (supporting). **Hongxin Niu:** Conceptualization (lead). **Ying Tang:** Conceptualization (equal); supervision (equal). **Lili Zhou:** Conceptualization (lead); funding acquisition (lead); resources (lead); supervision (lead); writing – review and editing (lead).

## FUNDING INFORMATION

This work was supported by National Natural Science Foundation of China (grant nos. 82225010, 82070707), the National Key R&D Program of China (2021YFC2500200), Outstanding Youths Development Scheme of Nanfang Hospital, Southern Medical University (2019J013, 2021J001), the Presidential Foundation of Nanfang Hospital (grant no. 2019Z006), Guangdong Provincial Clinical Research Center for Kidney Disease (no. 2020B1111170013), and Key Technologies R&D Program of Guangdong Province (2023B1111030004).

## CONFLICT OF INTEREST STATEMENT

The authors confirm that there are no conflicts of interest.

## Data Availability

The raw data supporting the conclusion of this article will be made available by the authors, without undue reservation.
